# The Reduction of Visceral Adipose Tissue after Roux-en-Y Gastric Bypass Is more Pronounced in Patients with Impaired Glucose Metabolism

**DOI:** 10.1007/s11695-018-3455-x

**Published:** 2018-08-14

**Authors:** Lucie Favre, Laura Marino, Aline Roth, James Acierno, Didier Hans, Nicolas Demartines, Nelly Pitteloud, Michel Suter, Tinh-Hai Collet

**Affiliations:** 1 Service of Endocrinology, Diabetes and Metabolism, Lausanne University Hospital, Rue Saint-Martin 3, 1003 Lausanne, Switzerland; 2 Centre of Bone Diseases, Bone and Joint Department, Lausanne University Hospital, Lausanne, Switzerland; 3 Department of Visceral Surgery, Lausanne University Hospital, Lausanne, Switzerland; 4 Department of Surgery, Riviera-Chablais Hospital, Aigle-Monthey, Switzerland

**Keywords:** Bariatric surgery, Roux-en-Y gastric bypass, Visceral adipose tissue, Type 2 diabetes, Dual-energy X-ray absorptiometry (DXA)

## Abstract

**Purpose:**

Visceral adipose tissue (VAT) is associated with cardiometabolic risk factors and insulin resistance. The physiological mechanisms underlying the benefits of Roux-en-Y gastric bypass surgery (RYGB) on glucose metabolism remain incompletely understood. The impact of RYGB on VAT was assessed among three groups of patients stratified by their glucose tolerance before surgery.

**Methods:**

Forty-four obese women were categorized into normoglycemia (*n* = 21), impaired glucose tolerance (IGT, *n* = 18) and diabetes (*n* = 5) before surgery. Body composition measured by dual-energy X-ray absorptiometry (DXA) was performed before surgery, 6 months and 12 months after.

**Results:**

The three groups had comparable mean age (mean 38.6 ± SD 9.9) and BMI at baseline (41.9 ± 4.3 kg/m^2^). After 12 months, total weight loss (mean 35.1% ± 7.5) and excess weight loss (91.1% ± 25.1) were similar between groups. Pre-surgery mean VAT was significantly higher in diabetes (mean 2495 ± 616 g) than in normoglycemia (1750 ± 617 g, *p* = 0.02). The percentage of VAT to total body fat was significantly higher in diabetes (mean 4.4% ± 0.9) compared to normoglycemia (2.9% ± 0.8, *p* = 0.003). Twelve months after surgery, VAT loss was significantly greater among patients with diabetes (mean 1927 ± 413 g) compared to normoglycemia (1202 ± 450, *p* = 0.009).

**Conclusions:**

RYGB leads to important VAT loss, and this loss is greater in patients with diabetes prior to surgery. As VAT is associated with insulin resistance, this reduction may account for the profound impact of this surgery on glucose metabolism.

## Introduction

In recent years, bariatric surgery has been shown to be the most effective strategy to achieve a significant and sustained long-term weight loss in severely obese patients [1]. Obesity is associated with an increased risk of metabolic comorbidities including type 2 diabetes. Bariatric surgery leads to a clear improvement in type 2 diabetes [2–4] and lowers the incidence of diabetes [5]. However, the physiological mechanisms underlying the impact of bariatric surgery on glucose metabolism are not fully understood [6]. Caloric restriction, proximal intestinal nutrient exclusion, rapid distal gut nutrient delivery [7], incretin secretion [8], changes in gut microbiota, and bile acid metabolism [9] are some of the suggested mechanisms involved in diabetes remission after bariatric surgery.

Fat distribution has important associations with diabetes and the risk of cardiovascular diseases. Abdominal fat has long been associated with insulin resistance and increased cardiovascular risk [10, 11]. In contrast, gluteofemoral fat may be protective for the development of these conditions [12]. Abdominal fat is composed of abdominal subcutaneous fat and intra-abdominal fat, also called visceral fat or visceral adipose tissue (VAT). This fat compartment primarily consists of omental and mesenteric fat, and directly drains through portal circulation into the liver [13]. VAT surrounds the internal organs and is associated with higher cardiometabolic risk and insulin resistance [14]. It is increased in patients with impaired glucose tolerance (IGT) or type 2 diabetes compared to patients with normal glucose tolerance [15]. VAT histology has shown cell stress, degeneration and necrosis in diabetic patients [16]. It has also been demonstrated that weight loss provides a decrease in hepatic and pancreatic fat that is associated with normalization in insulin sensitivity [17]. Ponti et al. studied changes in body composition measured by dual-energy X-ray absorptiometry (DXA) during weight loss programs in 48 obese women [18]. The modest weight reduction of 4.5% over 12 months of follow-up was accompanied by a marked reduction of 12% in VAT. In their recent article, Chooi et al. described the effect of a 5% diet-induced weight loss on body composition in 11 metabolically unhealthy lean Asian individuals (mean baseline BMI 22.7 kg/m^2^) [19]. Body composition and VAT were assessed by magnetic resonance (MR) imaging. The weight loss achieved in 6 to 16 weeks resulted in a reduction of total fat mass by 9%, reduction of VAT by 11% and in an increase in peripheral insulin sensitivity.

Considering the profound influence of VAT on glucose metabolism, we aimed to investigate the impact of weight loss after RYGB on body composition and VAT, as measured by DXA among patients stratified by their glucose tolerance before surgery.

## Methods

### Study Design and Population

After approval by the local institutional review board (decision 304/15), all consecutive female patients seen in our clinical pathway for RYGB between January 2015 and November 2016 were prospectively invited to participate in this study. During this period, 67 female patients underwent RYGB at our hospital, of which 44 (66%) enrolled in this observational study. Per local health regulations, adult patients were eligible for RYGB if they had a body mass index (BMI) ≥ 35.0 kg/m^2^ and had failed in previous conservative attempts at sustained weight loss. The exclusion criteria were type 1 diabetes, secondary diabetes or previous bariatric surgery. All patients were assessed and followed by a multidisciplinary team including an endocrinologist, psychiatrist, psychologist, nutritionist and a bariatric surgeon specialized in standardized management of obese individuals. Patients underwent laparoscopic RYGB with creation of a 15–20-ml gastric pouch, a 100–150-cm Roux limb, and a 30–50-cm biliopancreatic limb.

### Clinical and Biological Data

All measurements were made before, 6 months and 12 months after RYGB. Participants were weighed barefoot in light clothing to the nearest 0.1 kg. Height was measured with a fixed wall stapediometer. The % excess weight loss (EWL) was defined as the operative weight minus the follow-up weight, divided by the excess weight, and multiplied by 100. Excess weight was the operative weight minus ideal body weight based on a BMI of 25 kg/m^2^. The % total weight loss (TWL) was defined as the operative weight minus the follow-up weight, divided by the operative weight, and multiplied by 100.

Standard biological assays on fresh blood samples were measured at the Clinical Laboratory facility of the Lausanne University Hospital. Plasma glucose and glycated hemoglobin (HbA1c) were measured in all patients at baseline and 12 months post-surgery. Fasting insulin and 75-g oral glucose tolerance test (OGTT) was performed in non-diabetic patients prior to surgery. Insulin resistance (IR) index was determined using the homeostasis model assessment on insulin resistance (HOMA-IR) calculated according to the formula: HOMA-IR = fasting plasma glucose fasting insulin × 22.5 [20].

### Group Stratification

Patients were categorized before surgery into normoglycemia (reference group), IGT, and diabetes according to the current ADA definitions [21]. IGT was defined as fasting plasma glucose (FPG) between 5.6 and 6.9 mmol/L (between 100 and 125 mg/dL), or as 2-hour plasma glucose after 75-g OGTT levels between 7.8 and 11.0 mmol/L (between 140 and 199 mg/dL), or HbA1c between 5.7 and 6.4%. Diabetes was defined as FPG ≥ 7.0 mmol/L (≥ 126 mg/dL) or 2-hour plasma glucose ≥ 11.1 mmol/L (≥ 200 mg/dL) during the 75-g OGTT or HbA1c ≥ 6.5%.

### DXA and Analyses of the VAT Compartment

Whole-body DXA (GE Healthcare Lunar iDXA) was performed before, 6 and 12 months after surgery. The principles of the DXA methodology provide a 2-compartment measurement of fat and fat-free mass on the molecular level. Because of the limitation of the scanning area, an automated half-scan methodology to estimate whole-body composition from a half-body scan was used in 33 patients (75%) at baseline. This method has been validated for the analysis of whole-body analysis for fat mass, non-bone lean mass and percent fat [22].

VAT analysis of the android region was performed by the CoreScan™ software. For measuring android fat with iDXA, a region-of-interest was automatically defined between the top of the iliac crest, and 20% of the distance from the top of the iliac crest to the base of the skull. This region contains both subcutaneous and VAT, and is not subject to the half-body analysis. X-ray attenuation in the iDXA soft tissue image allows to determine the edge of the body and the outer edge of the abdominal cavity which is lighter due to the lower % of fat because of the muscles of the abdominal wall. VAT is computed by subtracting subcutaneous fat from the total android fat mass in the android region [23].

This software has been proven to be highly reliable for the measurement of VAT when compared to CT or MRI. Kaul et al. [24] studied 124 adults with BMI 18.5–40.0 kg/m^2^ and measured their VAT with both CT and DXA. The coefficient of determination (*r*^2^) for regression of CT on DXA values was 0.957. Reinhardt et al. [25] compared VAT measurements between iDXA and MRI in 40 patients and reported a coefficient of determination (*r*^2^) for regression of MRI on iDXA of 0.948.

### Statistical Analyses

Data are presented as mean ± standard deviation (SD) and were analyzed using Stata software (version 15.1, StataCorp, College Station, TX). The overall comparisons of continuous variables across the 3 patient groups were assessed with analysis of variance (ANOVA) and linear regression, with normoglycemic patients as the reference group. Due to the small number of patients, comparisons were not adjusted, unless stated otherwise. A two-sided *p*-value of 0.05 was considered significant.

## Results

### Baseline Anthropometric and Metabolic Characteristics

A total of 44 women within the RYGB clinical pathway between 2015 and 2017 were enrolled. The mean age at surgery was not different between those with normoglycemia, IGT, and diabetes (Table 1). Baseline BMI was lower in patients with diabetes (mean 39.9 ± 6.4 kg/m^2^) compared to those with IGT (mean 41.7 ± 3.5 kg/m^2^) and normoglycemia (mean 42.5 ± 4.4 kg/m^2^), albeit not significantly. In normoglycemia, baseline FPG was 5.0 ± 0.3 mmol/L (90 ± 5 mg/dL), 2-hour OGTT plasma glucose 6.2 ± 1.2 mmol/L (112 ± 22 mg/dL), HbA1c 5.1% ± 0.4, and HOMA-IR 4.8 ± 1.9. In IGT, baseline FPG was 5.6 ± 0.7 mmol/L (101 ± 13 mg/dL), 2-hour OGTT plasma glucose 7.8 ± 2.2 mmol/L (141 ± 40 mg/dL), HbA1c 5.4% ± 0.5, and HOMA-IR 6.9 ± 3.2. Patients with diabetes were all treated with oral antidiabetics at baseline, and two were additionally on insulin. The group’s mean HbA1c was 7.0% ± 0.4 before surgery.

**Table 1:** Age, evolution of weight, and BMI before, 6 months and 12 months post-RYGB

Mean ± SD	Normoglycemia (*n* = 21)	IGT (*n* = 18)	Diabetes (*n* = 5)	*P* for overall comparison
Age at surgery	37.2 ± 9.5	38.5 ± 11.2	44.9 ± 3.8	0.30
Weight (kg)				
Before surgery	112.6 ± 16.3	110.7 ± 12.4	115.0 ± 20.8	0.84
6 months post-op	80.3 ± 13.5	80.4 ± 13.4	83.3 ± 13.7	0.90
12 months post-op	71.6 ± 15.2	73.4 ± 14.6	77.6 ± 14.1	0.71
BMI (kg/m^2^)				
Before surgery	42.5 ± 4.4	41.7 ± 3.5	39.9 ± 6.4	0.47
6 months post-op	30.3 ± 4.1	30.2 ± 4.0	28.9 ± 4.2	0.78
12 months post-op	27.0 ± 4.9	27.5 ± 4.4	26.9 ± 4.4	0.92
Change in 12 months				
Weight loss (kg)	41.0 ± 10.8	37.3 ± 6.9	37.4 ± 8.2	0.41
Excess weight loss (%)	92.1 ± 24.4	88.5 ± 26.3	95.6 ± 28.6	0.83
Total weight loss (%)	36.6 ± 8.2	34.2 ± 7.4	32.5 ± 3.5	0.44

Abbreviations: BMI, body mass index; IGT, impaired glucose tolerance; RYGB, Roux-en-Y gastric bypass surgery

### Changes of Weight and Glucose Metabolism After Surgery

The overall weight loss and decrease in BMI after surgery were similar across groups, with EWL ranging from 35 to 139%, and TWL from 17 to 52% (Table 1). While those with normoglycemia had no change in their HbA1c levels 12 months after surgery, a small but statistically significant change was found in the HbA1c levels in IGT (mean −0.36% ± 0.48, *p* = 0.01 compared to normoglycemia). As expected, HbA1c was markedly decreased among patients with diabetes (mean 5.6% ± 0.2 after 1 year). Clinicians could therefore stop diabetes medication in all but one patient at 3 months, and after 10 months in the remaining one.

### Changes in Body Composition After Surgery

Body composition was measured with DXA at baseline, 6 months and 12 months after surgery (Table 2), except for six patients with normoglycemia who missed their appointment to the 6-month DXA. There was a marked reduction in total FM (−33.2 ± 8.8 kg) and regional body fat from baseline to 12-month follow-up for all patients, with no significant difference between groups. The average loss of LM over 12 months was 5.63 ± 2.66 kg across all groups, in addition to a limited decrease of bone mineral content (0.11 ± 0.17 kg).

**Table 2:** Body composition compartments from derived DXA indices, before and 12 months after RYGB

Mean ± SD	Normoglycemia (n = 21)		IGT (*n* = 18)		Diabetes (*n* = 5)		*P* for comparisons*
	Before surgery	12 months	Before surgery	12 months	Before surgery	12 months	
Total							
FM (kg)	59.78 ± 10.43	24.78 ± 10.18	57.07 ± 8.73	26.07 ± 9.94	57.31 ± 14.73	23.91 ± 8.05	
LM (kg)	49.08 ± 6.96	44.11 ± 6.78	50.46 ± 6.92	44.36 ± 6.43	56.22 ± 5.57	49.55 ± 4.58	0.12 / 0.04
Fat proportion (%)	54.8 ± 3.5	34.9 ± 8.3	53.0 ± 4.3	36.0 ± 8.9	49.9 ± 5.0	31.8 ± 5.8	0.05 / 0.02
Trunk							
FM (kg)	31.14 ± 6.36	11.38 ± 5.31	29.95 ± 5.01	12.49 ± 5.30	34.95 ± 7.60	12.53 ± 4.32	
LM (kg)	22.26 ± 2.96	21.55 ± 3.14	22.31 ± 3.14	20.95 ± 3.46	26.30 ± 2.81	24.98 ± 2.02	0.03 / 0.01
Fat proportion (%)	58.0 ± 3.7	33.1 ± 10.5	57.2 ± 3.6	36.1 ± 10.5	56.7 ± 4.0	32.6 ± 7.0	
Android							
FM (g)	5963 ± 1372	2086 ± 1576	5699 ± 1154	2110 ± 1023	6755 ± 1709	2156 ± 852	
LM (g)	3617 ± 475	3567 ± 843	3632 ± 552	3199 ± 635	4361 ± 616	4015 ± 499	0.02 / 0.007
Fat proportion (%)	54.4 ± 4.4	37.6 ± 7.9	52.5 ± 5.2	37.5 ± 8.9	49.1 ± 3.3	34.5 ± 5.0	0.07 / 0.03
VAT mass (g)	1750 ± 617	548 ± 283	1940 ± 601	502 ± 257	2495 ± 616	568 ± 268	0.06 / 0.02
VAT volume (cm^3^)	1784 ± 555	581 ± 299	1990 ± 591	532 ± 272	2645 ± 653	603 ± 284	0.02 / 0.005
SAT mass (g)	4213 ± 1046	1538 ± 1410	3759 ± 956	1608 ± 838	4260 ± 1346	1587 ± 617	
Gynoid							
FM (g)	10,446 ± 1784	4412 ± 1759	9482 ± 1994	4353 ± 1773	8631 ± 3249	3809 ± 1535	
LM (g)	7960 ± 1375	6785 ± 1210	8065 ± 1367	6565 ± 1053	8826 ± 1250	7470 ± 957	
Fat proportion (%)	56.7 ± 3.8	38.4 ± 7.8	53.8 ± 5.5	38.7 ± 8.9	48.3 ± 7.3	32.8 ± 6.5	0.005 / 0.002
Arms							
FM (g)	6113 ± 1110	2723 ± 1041	6731 ± 1406	3060 ± 1263	5847 ± 943	2714 ± 790	
LM (g)	5164 ± 1120	4368 ± 676	6033 ± 1048	4805 ± 752	6026 ± 411	4385 ± 1529	0.03 / 0.10
Fat proportion (%)	54.4 ± 4.5	37.3 ± 7.6	52.5 ± 5.2	37.6 ± 9.3	49.1 ± 3.3	34.7 ± 4.9	
Legs							
FM (kg)	21.62 ± 4.70	9.91 ± 4.15	19.46 ± 4.68	9.71 ± 3.94	15.52 ± 6.59	7.86 ± 3.49	0.05 / 0.02
LM (kg)	18.72 ± 3.32	15.42 ± 3.04	19.09 ± 2.94	15.69 ± 2.57	20.68 ± 2.44	16.68 ± 2.07	
Fat proportion (%)	53.4 ± 4.5	38.1 ± 7.4	50.1 ± 6.7	37.1 ± 8.6	41.5 ± 8.8	31.0 ± 8.1	0.001 / <0.001
Indices							
AFM/GFM	1.09 ± 0.07	0.86 ± 0.19	1.14 ± 0.11	0.95 ± 0.23	1.26 ± 0.13	1.04 ± 0.21	0.004 / 0.001
TFM/TLM	1.22 ± 0.18	0.56 ± 0.21	1.14 ± 0.19	0.59 ± 0.19	1.01 ± 0.20	0.48 ± 0.12	0.06 / 0.03
FMI (kg/m^2^)	23.1 ± 4.3	9.5 ± 3.8	21.8 ± 3.1	10.0 ± 3.6	20.2 ± 4.7	8.4 ± 2.7	
ALMI (kg/m^2^)	9.1 ± 1.0	7.5 ± 0.9	9.5 ± 1.1	7.8 ± 0.9	9.4 ± 0.9	7.7 ± 1.0	
VAT/SAT mass	0.42 ± 0.15	0.64 ± 0.62	0.56 ± 0.26	0.36 ± 0.23	0.62 ± 0.20	0.35 ± 0.11	

Abbreviations: AFM, android fat mass; ALMI, appendicular lean mass index (Appendicular lean mass / squared height); DXA, dual-energy X-ray absorptiometry; FM, fat mass; FMI, fat mass index (total body fat / squared height); GFM, gynoid fat mass; IGT, impaired glucose tolerance; LM, lean mass (excluding bone); Post-op, Post-operative (12 months after surgery); Pre-op, Pre-operative (before surgery); RYGB, Roux-en-Y gastric bypass surgery; SAT, subcutaneous adipose tissue; TFM, total body fat mass; TLM, total body lean mass; VAT, visceral adipose tissue

*The first *p*-value is the *P* for overall comparison across all groups, followed by the *P* for comparison between diabetes and normoglycemia. All comparisons were made with pre-op values. For legibility, non-significant *p*-values are not shown

Fat mass in the trunk was delineated by the software to separate the android and gynoid regions. In patients with diabetes, fat mass was disproportionally distributed within the android region compared to the gynoid region (A/G ratio 1.26 ± 0.13 in diabetes vs 1.09 ± 0.07 in normoglycemia, *p* < 0.001).

**Fig. 1:**
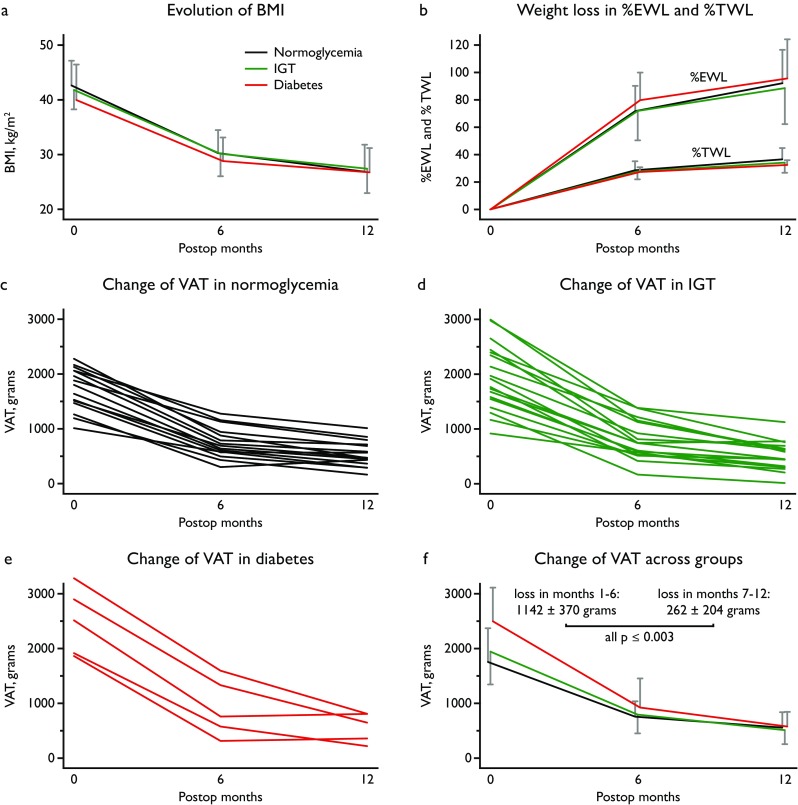
Weight loss and VAT evolution after surgery. Abbreviations: BMI, body mass index; DXA, dual-energy X-ray absorptiometry; %EWL, % excess weight loss; IGT, impaired glucose tolerance; %TWL, % total weight loss; VAT, visceral adipose tissue. Legend: Weight loss after Roux-en-Y gastric bypass surgery across the three groups (color legend shown in panel A) expressed as BMI (panel A), %EWL, and %TWL (panel B). Evolution of VAT mass as measured by DXA over time after surgery in each group (panels C-E) and comparison between groups (panel F). The 6-month DXA was missed by 6 patients with normoglycemia and their individual course is not shown in Panel D. The error bars represent SD

The changes in VAT over time were then specifically analyzed (Table 3). Pre-surgery VAT mass was significantly higher in diabetes (mean 2495 ± 616 g) than in normoglycemia (mean 1750 ± 617 g; overall *p* for comparison = 0.059, diabetes vs normoglycemia *p* = 0.02, Fig. 1). After surgery, VAT decreased across all groups to 531 g ± 266 after 12 months: VAT loss was significantly higher among patients with diabetes (mean 1927 g ± 413) compared to normoglycemia (mean 1203 g ± 461; overall *p* = 0.009 for comparison, diabetes vs normoglycemia *p* = 0.003). VAT loss was associated with HbA1c reduction across all groups (linear regression *p* = 0.02). VAT loss occurred relatively quickly within the first 6 months after surgery (mean 1142 ± 370 g), while this effect seemed to slow from months 7 to 12 (mean 262 ± 204 g) across all groups (all *p* ≤ 0.003). VAT in proportion to total body fat was significantly increased in diabetes (mean 4.4% ± 0.9) compared to normoglycemia (mean 2.9% ± 0.8; overall *p* for comparison = 0.007, diabetes vs normoglycemia *p* = 0.002). Pre-surgery VAT mass was correlated with HbA1c (Pearson rho = 0.32), fasting insulin (Pearson rho = 0.35) and HOMA-IR (Pearson rho = 0.37; linear regression: unadjusted *p* = 0.03, age and BMI-adjusted *p* ≤ 0.01).

**Table 3:** Evolution of VAT before, 6 months and 12 months post-RYGB

Mean ± SD	Normoglycemia (*n* = 21)	IGT (*n* = 18)	Diabetes (*n* = 5)	*P* for overall comparison	IGT vs normoglycemia	Diabetes vs normoglycemia
VAT (g)						
Before surgery	1750 ± 617	1940 ± 601	2495 ± 616	0.059	*p* = 0.34	*p* = 0.02
6 months post-op*	749 ± 281	787 ± 344	916 ± 533	0.65		
12 months post-op	548 ± 283	502 ± 257	568 ± 268	0.83		
VAT/total body fat (%)						
Before surgery	2.90 ± 0.80	3.45 ± 1.08	4.43 ± 0.90	0.007	*p* = 0.08	*p* = 0.002
6 months post-op*	2.28 ± 0.55	2.42 ± 0.86	2.98 ± 1.04	0.23		
12 months post-op	2.28 ± 0.95	1.93 ± 0.84	2.33 ± 0.79	0.42		
Changes in 12 months						
VAT loss (g)	1202 ± 450	1438 ± 470	1927 ± 413	0.009	*p* = 0.11	*p* = 0.003
VAT change (%)	−69.2 ± 11.1	−74.6 ± 10.4	−78.0 ± 7.6	0.14		
VAT/total body fat (%)	−0.63 ± 1.09	−1.52 ± 0.67	−2.09 ± 0.60	0.001	*p* = 0.003	*p* = 0.002

Abbreviations: DXA, dual-energy X-ray absorptiometry; IGT, impaired glucose tolerance; RYGB, Roux-en-Y gastric bypass surgery; VAT, visceral adipose tissue

*VAT was assessed in only 15 patients with normoglycemia at the 6-month DXA due to missed appointment

## Discussion

In this study of VAT evolution over 12 months after bariatric surgery, the impact of RYGB was larger among those with diabetes compared to those with IGT or normoglycemia. The three groups had comparable mean age and BMI at baseline, while pre-surgery VAT mass was significantly higher among patients with diabetes. The majority of VAT loss occurred rapidly within the first 6 months after surgery, while the effect slowed during months 7–12. Interestingly, VAT after 12 months was comparable across all groups, even in diabetes where they started with a large excess of VAT prior to surgery. This may relate to the better control of diabetes in this group where diabetes medications could be stopped in all but 1 patient at 3 months, although these observational data cannot conclude a causal link.

Overall weight loss and decrease in BMI after surgery were similar across groups, with EWL ranging from 35 to 139%, and TWL from 17 to 52%. These results are consistent with other reports on EWL and TWL 12 months post RYGB [26, 27]*.* Some studies showed that patients with diabetes lost less weight after bariatric surgery [28, 29]. Our study included only five patients with diabetes and their TWL was not significantly different from the other groups. The study sample size is too small to draw conclusions.

Few studies reported changes in body composition assessed by DXA after bariatric surgery. Tamboli et al. [30] studied 29 subjects before and up to 12 months after RYGB and showed that LM loss contributed to 10.2% of total weight loss during the first year after surgery, with this loss occurring primarily in the first post-operative month. Vatier et al*.* [31] also reported body composition changes assessed by DXA in 86 obese patients up to 12 months after RYGB. However, VAT mass and glucose metabolism parameters were not reported in these two studies.

We found a remarkable 72% reduction in VAT at 12 months post-surgery (−69% in normoglycemia, −75% in IGT, and −78% in diabetes). These results are in agreement with others studies which reported VAT loss assessed either by MR imaging or DXA. Toro-Ramos et al. reported a 77% VAT loss as measured by MR imaging 12 months after different types of bariatric surgery in 23 men and women [32]. Nine obese women studied by Johansson et al. experienced a mean VAT reduction of 73% 12 months after bariatric surgery (MR imaging) [33]. Bazzochi et al [34] showed a 65% reduction in VAT (DXA) (from 2660 ± 1560 g to 880 ± 730 g) among 29 women followed for 1 year after RYGB. The baseline metabolic characteristics of the patients were not reported in these studies. To our knowledge, the present study is the first to characterize body composition changes assessed via DXA with respect to glucose tolerance before surgery.

Not all obese individuals are insulin resistant, and fat mass per se is not the sole determinant of insulin resistance. Body fat distribution is recognized as an important predictor and modifier of the adverse health consequences of obesity such as T2DM, and VAT has been previously reported to be more prominent in patients with type 2 diabetes than in patients without [14, 35]. The present data report the same correlation with a higher VAT in proportion of EW in type 2 diabetes patients at baseline. Interestingly, the VAT mass in absolute terms and in proportion to the total fat mass was clearly greater in diabetes than in normoglycemia before surgery. This difference was not apparent 12 months after RYGB. This comparison should be repeated in other studies according to glucose tolerance before bariatric surgery.

While the changes in VAT were clear across groups, no other significant difference in the changes of fat mass distribution across body compartments was observed. In this present study, LM decreased by 11% after RYGB. This is comparable with reports of a 10% decline in initial LM [34] and 14% [31], but lower than in another study reporting 19% decline in LM post-surgery [30]. As previously described, the loss of LM occurred mostly during the first 6 months after RYGB (mean loss 5.37 ± 2.12 kg) [30, 34]. During months 7–12, the LM decreased only slightly (mean 0.43 ± 1.35 kg) with no significant differences observed across the groups.

Interestingly, Kashyap et al. [36] compared the body composition evolution of 18 subjects who underwent RYGB and 19 sleeve gastrectomies for 24 months. They observed that despite a similar weight loss, the loss of trunk fat was greater in the RYGB versus the sleeve gastrectomy group. However, VAT was not assessed in their study. It would be of interest to further compare RYGB and sleeve gastrectomy concerning this particular point, as sleeve gastrectomy has recently become the most commonly performed procedure in several countries [37–39].

### Limitations/Strengths

The present study was deliberately limited to women, due to the low number of men in our clinical pathway. However, this allowed a decreased heterogeneity of fat mass distribution, which has been well described between both sexes [40]. Given the small population size and the low number of patients with diabetes before surgery, the results of this study should be regarded as preliminary. Future studies are needed to increase the number and strengthen the suggestion that VAT loss may contribute to the improvement of glucose metabolism after RYGB. Moreover, this study was conducted in white European patients and precludes the generalization to other populations in order to determine ethnicity-specific differences in VAT evolution after RYGB.

## Conclusions

Adipose tissue plays a central role in the pathogenesis of obesity-induced insulin resistance [41]. The mechanisms underlying the profound impact of RYGB on glucose metabolism are not yet fully understood, and a reduction in VAT may account for a crucial role. The amount of weight loss after bariatric surgery is not different between patients with diabetes and normoglycemia, but this study demonstrated that the reduction in VAT was significantly larger in diabetes. Because metabolic comorbidities may occur at different BMI thresholds based on ethnic, sex and genetic characteristics, adopting a strictly BMI-based criterion for bariatric surgery has been challenged in recent years. As the assessment of VAT by DXA involves a small radiation dose and is not costly, it may become an important criterion for metabolic surgery in the future.

## References

[1] Adams TD, Davidson LE, Litwin SE, Kim J, Kolotkin RL, Nanjee MN, Gutierrez JM, Frogley SJ, Ibele AR, Brinton EA, Hopkins PN, McKinlay R, Simper SC, Hunt SC (2017). Weight and metabolic outcomes 12 years after gastric bypass. N Engl J Med.

[2] Schauer PR, Bhatt DL, Kirwan JP, Wolski K, Aminian A, Brethauer SA, Navaneethan SD, Singh RP, Pothier CE, Nissen SE, Kashyap SR, STAMPEDE Investigators (2017). Bariatric surgery versus intensive medical therapy for diabetes - 5-year outcomes. N Engl J Med.

[3] Sjostrom L, Lindroos AK, Peltonen M, Torgerson J, Bouchard C (2004). Lifestyle, diabetes, and cardiovascular risk factors 10 years after bariatric surgery. N Engl J Med.

[4] Mingrone G, Panunzi S, De Gaetano A, Guidone C, Iaconelli A, Nanni G (2015). Bariatric–metabolic surgery versus conventional medical treatment in obese patients with type 2 diabetes: 5 year follow-up of an open-label, single-centre, randomised controlled trial. Lancet.

[5] Vest AR, Heneghan HM, Agarwal S, Schauer PR, Young JB (2012). Bariatric surgery and cardiovascular outcomes: a systematic review. Heart.

[6] Batterham RL, Cummings DE (2016). Mechanisms of diabetes improvement following bariatric/metabolic surgery. Diabetes Care.

[7] Breen DM, Rasmussen BA, Kokorovic A, Wang R, Cheung GW, Lam TK (2012). Jejunal nutrient sensing is required for duodenal-jejunal bypass surgery to rapidly lower glucose concentrations in uncontrolled diabetes. Nat Med.

[8] Laferrere B, Heshka S, Wang K, Khan Y, McGinty J, Teixeira J, Hart AB, Olivan B (2007). Incretin levels and effect are markedly enhanced 1 month after Roux-en-Y gastric bypass surgery in obese patients with type 2 diabetes. Diabetes Care.

[9] Patti ME, Houten SM, Bianco AC, Bernier R, Larsen PR, Holst JJ, Badman MK, Maratos-Flier E, Mun EC, Pihlajamaki J, Auwerx J, Goldfine AB (2009). Serum bile acids are higher in humans with prior gastric bypass: potential contribution to improved glucose and lipid metabolism. Obesity.

[10] Despres JP, Lemieux I (2006). Abdominal obesity and metabolic syndrome. Nature.

[11] Tchernof A, Despres JP (2013). Pathophysiology of human visceral obesity: an update. Physiol Rev.

[12] Snijder MB, Dekker JM, Visser M, Bouter LM, Stehouwer CD, Yudkin JS, Heine RJ, Nijpels G, Seidell JC, Hoorn study (2004). Trunk fat and leg fat have independent and opposite associations with fasting and postload glucose levels. Diabetes Care.

[13] Wajchenberg BL (2000). Subcutaneous and visceral adipose tissue: their relation to the metabolic syndrome. Endocr Rev.

[14] Smith JD, Borel AL, Nazare JA, Haffner SM, Balkau B, Ross R, Massien C, Alméras N, Després JP (2012). Visceral adipose tissue indicates the severity of cardiometabolic risk in patients with and without type 2 diabetes: results from the INSPIRE ME IAA study. J Clin Endocrinol Metab.

[15] Borel AL, Nazare JA, Smith J, Aschner P, Barter P, Van Gaal L (2015). Visceral, subcutaneous abdominal adiposity and liver fat content distribution in normal glucose tolerance, impaired fasting glucose and/or impaired glucose tolerance. Int J Obes.

[16] Camastra S, Vitali A, Anselmino M, Gastaldelli A, Bellini R, Berta R, Severi I, Baldi S, Astiarraga B, Barbatelli G, Cinti S, Ferrannini E (2017). Muscle and adipose tissue morphology, insulin sensitivity and beta-cell function in diabetic and nondiabetic obese patients: effects of bariatric surgery. Sci Rep.

[17] Lim EL, Hollingsworth KG, Aribisala BS, Chen MJ, Mathers JC, Taylor R (2011). Reversal of type 2 diabetes: normalisation of beta cell function in association with decreased pancreas and liver triacylglycerol. Diabetologia.

[18] Ponti F, Soverini V, Plazzi A, Aparisi Gomez MP, Mercatelli D, Guglielmi G (2018). DXA-assessed changes in body composition in obese women following two different weight loss programs. Nutrition.

[19] Chooi YC, Ding C, Chan Z, Choo J, Sadananthan SA, Michael N, Lee Y, Velan SS, Magkos F (2018). Moderate weight loss improves body composition and metabolic function in metabolically unhealthy lean subjects. Obesity.

[20] Matthews DR, Hosker JP, Rudenski AS, Naylor BA, Treacher DF, Turner RC (1985). Homeostasis model assessment: insulin resistance and fl-cell function from fasting plasma glucose and insulin concentrations in man. Diabetologia.

[21] American Diabetes A (2017). Classification and diagnosis of diabetes. Diabetes Care.

[22] Rothney MP, Brychta RJ, Schaefer EV, Chen KY, Skarulis MC (2009). Body composition measured by dual-energy X-ray absorptiometry half-body scans in obese adults. Obesity.

[23] Micklesfield Lisa K., Goedecke Julia H., Punyanitya Mark, Wilson Kevin E., Kelly Thomas L. (2012). Dual-Energy X-Ray Performs as Well as Clinical Computed Tomography for the Measurement of Visceral Fat. Obesity.

[24] Kaul S, Rothney MP, Peters DM, Wacker WK, Davis CE, Shapiro MD, Ergun DL (2012). Dual-energy X-ray absorptiometry for quantification of visceral fat. Obesity.

[25] Reinhardt M, Piaggi P, DeMers B, Trinidad C, Krakoff J (2017). Cross calibration of two dual-energy X-ray densitometers and comparison of visceral adipose tissue measurements by iDXA and MRI. Obesity.

[26] Duvoisin C, Favre L, Allemann P, et al. Roux-en-Y gastric bypass: ten-year results in a cohort of 658 patients. Ann Surg. 2017. 10.1097/SLA.0000000000002538. 10.1097/SLA.0000000000002538 29194086

[27] Courcoulas AP, Christian NJ, Belle SH, Berk PD, Flum DR, Garcia L, Horlick M, Kalarchian MA, King WC, Mitchell JE, Patterson EJ, Pender JR, Pomp A, Pories WJ, Thirlby RC, Yanovski SZ, Wolfe BM, Longitudinal Assessment of Bariatric Surgery (LABS) Consortium (2013). Weight change and health outcomes at 3 years after bariatric surgery among individuals with severe obesity. JAMA.

[28] Still CD, Wood GC, Chu X, Manney C, Strodel W, Petrick A, Gabrielsen J, Mirshahi T, Argyropoulos G, Seiler J, Yung M, Benotti P, Gerhard GS (2014). Clinical factors associated with weight loss outcomes after Roux-en-Y gastric bypass surgery. Obesity.

[29] Junior WS, do Amaral JL, Nonino-Borges CB (2011). Factors related to weight loss up to 4 years after bariatric surgery. Obes Surg.

[30] Tamboli RA, Hossain HA, Marks PA, Eckhauser AW, Rathmacher JA, Phillips SE, Buchowski MS, Chen KY, Abumrad NN (2010). Body composition and energy metabolism following Roux-en-Y gastric bypass surgery. Obesity.

[31] Vatier C, Henegar C, Ciangura C, Poitou-Bernert C, Bouillot JL, Basdevant A, Oppert JM (2012). Dynamic relations between sedentary behavior, physical activity, and body composition after bariatric surgery. Obes Surg.

[32] Toro-Ramos T, Goodpaster BH, Janumala I, Lin S, Strain GW, Thornton JC, Kang P, Courcoulas AP, Pomp A, Gallagher D (2015). Continued loss in visceral and intermuscular adipose tissue in weight-stable women following bariatric surgery. Obesity.

[33] Johansson L, Roos M, Kullberg J, Weis J, Ahlstrom H, Sundbom M (2008). Lipid mobilization following Roux-en-Y gastric bypass examined by magnetic resonance imaging and spectroscopy. Obes Surg.

[34] Bazzocchi A, Ponti F, Cariani S, Diano D, Leuratti L, Albisinni U, Marchesini G, Battista G (2015). Visceral fat and body composition changes in a female population after RYGBP: a two-year follow-up by DXA. Obes Surg.

[35] Sam S, Haffner S, Davidson MH, D'Agostino RB, Feinstein S, Kondos G (2008). Relationship of abdominal visceral and subcutaneous adipose tissue with lipoprotein particle number and size in type 2 diabetes. Diabetes.

[36] Kashyap SR, Bhatt DL, Wolski K, Watanabe RM, Abdul-Ghani M, Abood B, Pothier CE, Brethauer S, Nissen S, Gupta M, Kirwan JP, Schauer PR (2013). Metabolic effects of bariatric surgery in patients with moderate obesity and type 2 diabetes. Diabetes Care.

[37] Spaniolas K, Kasten KR, Brinkley J, Sippey ME, Mozer A, Chapman WH, Pories WJ (2015). The changing bariatric surgery landscape in the USA. Obes Surg.

[38] Burns EM, Naseem H, Bottle A, Lazzarino AI, Aylin P, Darzi A, Moorthy K, Faiz O (2010). Introduction of laparoscopic bariatric surgery in England: observational population cohort study. BMJ.

[39] Czernichow S, Paita M, Nocca D, Msika S, Basdevant A, Millat B (2016). Current challenges in providing bariatric surgery in France: A nationwide study. Medicine (Baltimore).

[40] Kautzky-Willer A, Harreiter J, Pacini G (2016). Sex and gender differences in risk, pathophysiology and complications of type 2 diabetes mellitus. Endocr Rev.

[41] Kahn SE, Cooper ME, Del Prato S (2014). Pathophysiology and treatment of type 2 diabetes: perspectives on the past, present, and future. Lancet.

